# Analysis of Common Pathways and Markers From Non-Alcoholic Fatty Liver Disease to Immune-Mediated Diseases

**DOI:** 10.3389/fimmu.2021.667354

**Published:** 2021-11-24

**Authors:** Rocío Gallego-Durán, Rocío Montero-Vallejo, Douglas Maya-Miles, Ana Lucena, Franz Martin, Javier Ampuero, Manuel Romero-Gómez

**Affiliations:** ^1^ SeLiver Group, Instituto de Biomedicina de Sevilla/Consejo Superior de Investigaciones Científicas (CSIC)/Hospital Virgen del Rocío, Sevilla, Spain; ^2^ Biomedical Research Network on Hepatic and Digestive Diseases (CIBEREHD), Instituto de Salud Carlos III, Madrid, Spain; ^3^ Digestive Diseases Unit, Hospital Universitario Virgen del Rocío, Sevilla, Spain; ^4^ Andalusian Center for Molecular Biology and Regenerative Medicine (CABIMER), University of Pablo de Olavide-University of Sevilla-Consejo Superior de Investigaciones Científicas, Sevilla, Spain; ^5^ Biomedical Research Network on Diabetes and Related Metabolic Diseases (CIBERDEM), Madrid, Spain

**Keywords:** NAFLD, NASH, immunology, fibrosis, biomarkers

## Abstract

Metabolic associated fatty liver disease (MAFLD) is the most prevalent form of liver disease worldwide, accounting for a high liver-related mortality and morbidity with extensive multi-organ involvement. This entity has displaced viral hepatitis as the main cause of severe forms of hepatic diseases, although the onset and transition of MAFLD stages still remains unclear. Nevertheless, innate and adaptive immune responses seem to play an essential role in the establishment and further progression of this disease. The immune system is responsible of safeguard and preserves organs and systems function, and might be altered under different stimuli. Thus, the liver suffers from metabolic and immune changes leading to different injuries and loss of function. It has been stablished that cell-cell crosstalk is a key process in the hepatic homeostasis maintenance. There is mounting evidence suggesting that MAFLD pathogenesis is determined by a complex interaction of environmental, genetic and host factors that leads to a full plethora of outcomes. Therefore, herein we will revisit and discuss the interplay between immune mechanisms and MAFLD, highlighting the potential role of immunological markers in an attempt to clarify its relationship.

## Background

Metabolic associated liver disease (MAFLD), formerly known as non-alcoholic fatty liver disease (NAFLD) (1) is the most prevalent liver disorder worldwide, accounting for a non-negligible 25% in the overall population (2). The MAFLD spectrum is very wide, ranging from simple steatosis, initially benign, to non-alcoholic steatohepatitis or NASH, characterized by inflammation and ballooning degeneration. On the other hand, different stages of liver fibrosis appear, in some cases evolving to cirrhosis, various types of hepatic decompensations and eventually hepatocellular carcinoma. Reciprocally, MAFLD is a risk factor for other metabolic diseases, such as cardiovascular disease. Currently, liver fibrosis is the best predictor of mortality among these patients (3), and additionally, it has been recently reported that significant fibrosis forecasts new onset of both diabetes mellitus and arterial hypertension in NASH patients (4). Moreover, all MAFLD histological stages have been associated with significantly increased overall mortality, and this risk increases stepwise with the worsening of the MAFLD histology (5).

Due to this blend of histological parameters and metabolic disarrangements, no effective therapeutic treatment has been already marketed for this disease. Identifying the most effective intervention in patients with MAFLD is challenging. One of the main difficulties relies in the stratification of patients, since the gold standard method is currently the liver biopsy. The two main components of histological evaluation of the disease are the activity and hepatic fibrosis (6). This procedure is widespread and has well-validated scores, being NAS Score (7) and SAF Score (8) most probably the more extended. Nevertheless, is not exempt of disadvantages, such as invasiveness, cost and inter- and intra-observer variability. Thus, bursts of non-invasive diagnostic and prognostic biomarkers have emerged during recent years trying to cover the handicaps associated to liver biopsy (9).

Taking into account the lack of novel molecules lack of novel molecules that can ameliorate this condition, the search of therapeutic treatments is currently booming. Assignment of the best strategy for intervention, mainly through the modification of lifestyle (10) or the inclusion in clinical trials for MAFLD that still offer suboptimal results remains unclear (11). Combination therapies based on the various pathophysiological driver events including macrophage recruitment, polarization and action might be an intriguing target for future investigation (12).

In recent years, mounting evidence from preclinical and clinical studies have pointed out the role of immune system in the pathophysiology of MAFLD. Among other factors, it has been stated that MAFLD is a direct outcome of the mixture of overnutrition together with a decrease in physical activity. This situation has been reported even in pediatric population, with evidence that the maternal diet and the early life nutritional environment are correlated with the susceptibility of MAFLD in offspring. In this regard, a recent preclinical study revealed that maternal high-fat diet leads to intrauterine inflammation, which subsequently caused transgenerationally abnormal hepatic lipid metabolism (13). Nevertheless, the mechanisms underlying this situation are still unraveled, but the main body of evidence points out the role of macrophage as accelerators of fibrogenesis, and gut microbiota as modulators of the naïve immune system (14). Finally, in this disease there is a substantial amount of innate and adaptive mechanisms involved in the onset of the chronic inflammation. Herein we revisit the underpinning MAFLD mechanisms from the immune system point of view.

## Mechanisms of Inflammation

**Figure 1:**
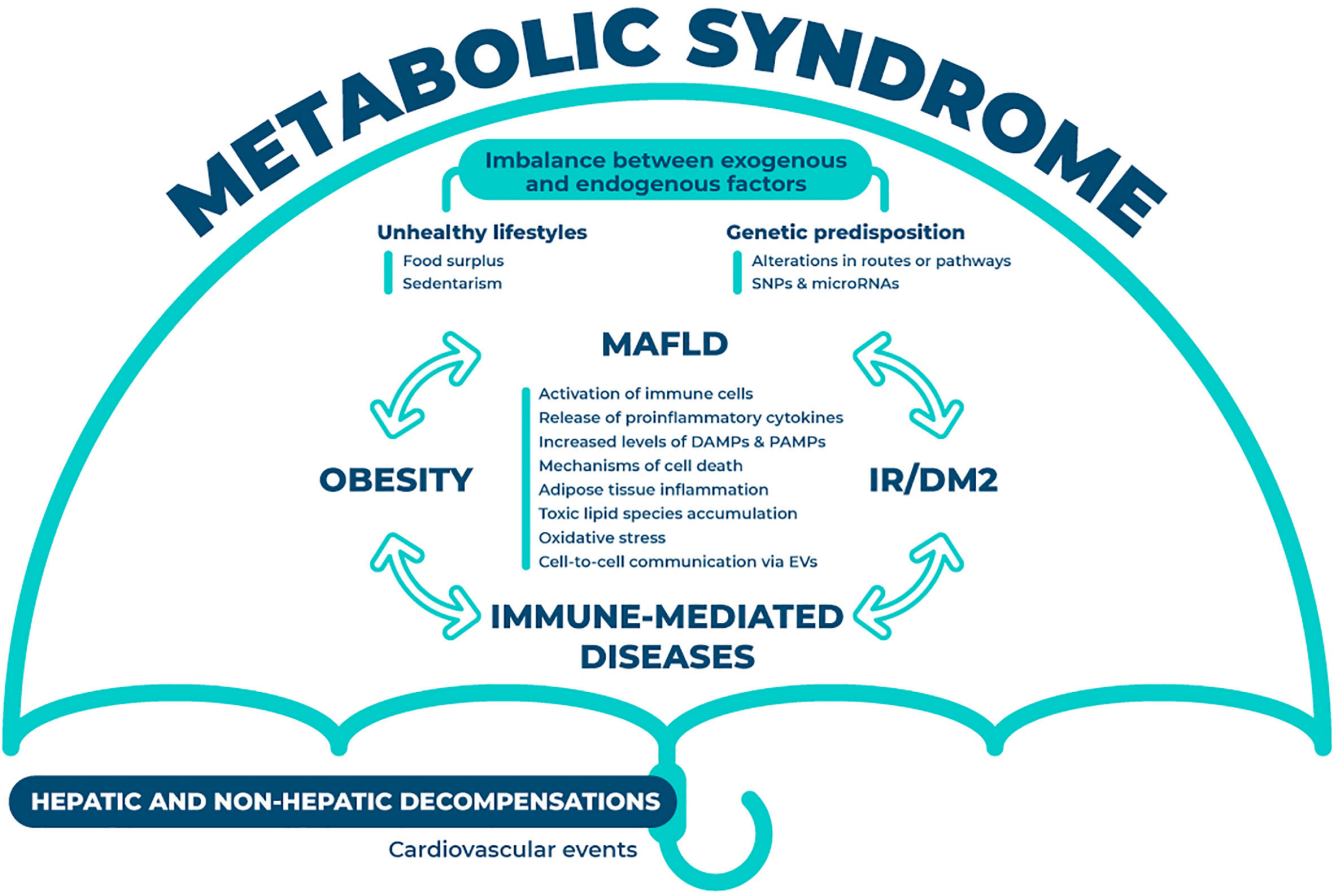
Interorgan crosstalk in MAFLD. The crosstalk between different organs and systems is summarized in this figure. Under the umbrella of metabolic syndrome, this disease is partially dictated through the imbalance between endogenous (i.e. genetic predisposition) and exogenous factors (i.e. unhealthy lifestyles). The interplay between MAFLD, insulin resistance or diabetes mellitus type 2, obesity and immune-mediated diseases is constant and occurs in all directions. This plethora of interactions will result in the aberrant activation of various mechanisms (i.e. immune cells, oxidative stress and cell death) that will eventually foster the hepatic and non-hepatic decompensations. MAFLD, metabolic associated fatty liver disease; SNPs, single nucleotide polymorphism; IR, insulin resistance; DM2, diabetes mellitus type 2; DAMPs, damage-associated molecular patterns; PAMPs, pathogen-associated molecular patterns; EVs, extracellular vesicles.

MAFLD is not an isolated condition but a multisystem situation, and it is considered the hepatic manifestation of the metabolic syndrome, affecting different organs and systems, such as the cardiovascular system (**Figure 1**). Although the exact interactions remain elusive, previous research has proven the role of the innate and the adaptive immune systems in the development and transition to non-alcoholic steatohepatitis. The multiple-hit hypothesis of MAFLD has been supported by recent findings, which states that systemic changes in the liver, intestinal tract, and/or adipose tissue lead to the development of MAFLD (15). Obesity and MAFLD are intimately linked due to the disturbed systemic energy balance characterized by food surplus that constitutes one of the classical contributing factors for this disease. Low-level of chronic inflammation is representative of obesity. Obesity changes the composition of immune cells in adipose tissue, triggering inflammation and adipocytes death, which in a vicious circle also aggravates obesity. Inflammation of adipose tissue results in a release of adipokines that increases the flux of free fatty acids and the infiltration of macrophages in the liver, also impelling MAFLD progression (16). This situation of sustained inflammation mediates insulin resistance and provides a link between both entities. Further, gut dysbiosis has also been associated to this disease, through the alteration of the mucosal barrier integrity, mediated by the translocation of bacterial products (17). Gut metabolites together with pathogen-associated molecular patterns (PAMPs) modulate the metabolic and immune response in different organs, including adipose tissue, muscle and liver (18). Finally, hepatic immune cells are also activated by the metabolic overload, more specifically through an excessive lipid accumulation that additionally contributes to the development of pathological signs.

Different mechanisms of hepatic inflammation that drive disease progression have been already identified. Liver cells of the innate immune system play a key role in the shift of the tolerogenic environment of the liver towards an immunogenic phenotype. This process is accomplished through the recognition of various signals, such as damage-associated molecular patterns (DAMPs), released from parenchymal and non –parenchymal hepatic cells, together with the recognition of PAMPs and bacterial products from enterohepatic circulation through pattern recognition receptors (PPRs) from immune cells (19). In recent years, these simultaneous exogenous and endogenous events have been under research in an attempt to develop novel and effective therapeutic targets and/or potential non-invasive biomarkers. Innate immune responses involved in NASH include the activation of Kupffer cells together with the recruitment of leukocytes, natural killer and natural killer T cells to the liver (20). On the other hand, adaptive immunity is acquired as a consequence of the exposure to external antigens through lifetime, generating a vast spectrum of specific receptors and immune memory.

Liver and intestine communicate in a bidirectional way, where the gut microbiota participates in the development of different liver diseases. Diet-induced metabolic endotoxemia is a significant cause of chronic diseases, where excessive intake of fructose can be crucial (21). Toll-like receptors (TLRs) are transmembrane plasma bound receptors that belong to the PPRs family, expressed in most liver cells. They are important receptors in the immune innate system as they detect the presence of microbial infections and are expressed by immune cells recognizing a variety of stimuli, to help initiate and promote the immune response (22). Each TLR recognizes specific pathogen components and initiates the response by producing inflammatory molecules. Indeed, the gut epithelium is a barrier to avoid the lipopolysaccharide (LPS) to be absorbed, but once the permeability of this defence is increased, LPS can translocate and enter into the bloodstream. LPS is a PAMP that activates TLR4, induces liver inflammation and participates in the development of hepatic fibrosis, and can be transported by bacterial extracellular vesicles, among others (23). Therefore, the translocation of LPS results in an increase in LPS plasmatic levels, followed by TLR4 activation (24). A recent study has proven the role of microbiota-derived TLR agonists in promoting hepatic steatosis without affecting fructose-1-phosphate and cytosolic acetyl-CoA, reinforcing the idea that endotoxin engages TLR4 to trigger tumor necrosis factor (TNF) production by liver macrophages (25). Finally, abolition of TLR9, a pattern recognition receptor which binds DNA derived from intestinal bacteria, leads to the attenuation of both steatohepatitis and liver fibrosis in a preclinical NASH model (26). In the set of systemic inflammatory molecules, the contribution of mineralocorticoid receptor (MR) is under investigation. In a recent study performed in methionine-choline deficient diet (MCD) animals it has been reported that proinflammatory cells are functionally suppressed in the absence of MR. This loss might modulate the adaptive immune response, eventually leading to a reduction in lipid accumulation in the liver (27).

CD (cluster of differentiation) are cell surface molecules expressed on leucocytes and other relevant cells for the immune system (28). Recent studies suggest that CD44 has an important role in the development and progression of MAFLD. CD44 is a multifunctional cell membrane protein expressed by most cell types, such as macrophages and hepatocytes, reported to regulate a variety of inflammatory responses, including the induction of proinflammatory cytokines and the migration of macrophages and neutrophils (29). Osteopontin, e-selectin and hyaluronic acid are the main ligands of CD44, and human adipose tissue CD44 is associated with localized inflammation and systemic insulin resistance (30). CD44 seems to play a key role in the progression from simple steatosis to NASH by regulating macrophage polarization and infiltration (31). Soluble CD163 is the ectodomain of the haemoglobin-haptoglobin scavenger receptor, which is particularly expressed on macrophages and monocytes. It is released to the circulation throughout macrophage activation by metalloprotease activity (32, 33). CD163-positive macrophages are strongly expressed in human adipose tissue from obese individuals, while CD163 levels are associated with hepatic inflammation and fibrosis in patients with MAFLD (34). These levels have proven to be decreased after bariatric surgery (35) and successful life-style interventions (36).

Besides, as stated before, MAFLD has been traditionally associated with an impaired hepatic mitochondrial homeostasis and worsened energetic metabolism. Single cell analysis of healthy and cirrhotic human livers has identified a novel scar-associated macrophage subpopulation expressing triggering receptor expressed on myeloid cells-2 (TREM2), which expanded in liver fiver fibrosis conditions (37). The TREM family includes five members, and among them TREM1 and TREM2 are expressed in the cell membrane where they receive and transmit external signals. TREM2 is expressed in non-parenchymal liver cells such as liver sinusoidal endothelial cells, Kupffer cells, and hepatic stellate cells (38). More specifically, in human hepatocellular carcinoma tumours, TREM2 was found to be expressed in infiltrating monocyte derived macrophages, and also in certain neutrophils and tissue-specific macrophages, including osteoclasts and microglia (39). TREM2 is upregulated during diverse situations of liver injury in humans and mice, promoting the resolution of inflammation by inhibiting the yield of pro-inflammatory factors and ultimately preventing parenchymal cell death (40), and negatively regulating the synthesis of cytokines, such as tumor necrosis factor-α (TNF-α) and interleukin-6 (IL-6) in macrophages and dendritic cells (41). Finally, in a large clinical cohort a link between mortality in MAFLD-associated sepsis was recently observed. Authors discovered a metabolic coordination between hepatocyte mitochondria and liver macrophages expressing TREM2, whereas macrophages lacking from Trem2 released exosomes that impaired hepatocytic mitochondrial structure and energy supply through the blockage of Mitofusin 2 by miR-106b-5p (42), positioning TREM2 as a potential therapeutic target.

A variety of hepatocyte cell death mechanisms have been recognized as key factors for NASH development involving different immune cells types, and take place in distinct pathophysiological processes. Apoptosis is the most studied mechanism of cell death and can be triggered either intrinsically, by mechanisms such as DNA damage or oxidative stress, or extrinsically, being initiated by perturbations of the extracellular microenvironment, mainly driven by the stimulation of transmembrane receptors or pattern recognition receptors (43). In the liver, apoptotic hepatocytes can initiate inflammation by activating macrophages, together with the release of cytokeratin 18 (CK-18), the main intermediate filament protein in hepatocytes. Raised circulating CK-18 levels, both M30 (caspase-cleaved CK-18) and M65 (full length protein) have been proposed as non-invasive biomarkers for NASH and fibrosis, alone or in composite-biomarkers. A recent metanalysis has described a better performance for M65 than M30 in NASH diagnosis (44). Additionally, lytic forms of hepatocellular death elicit strong inflammatory responses due to cell membrane permeabilization and the releasement of intracellular content, such as DAMPs, that contributes to recruitment of immune cells (45). Among them, pyroptosis is a specific mechanism of cell death that results in the formation of pores in the plasmatic membrane leading to the delivery of intracellular and inflammatory components from the hepatocyte into the extracellular space. From there, they can be phagocyted by neighbour cells, mediating inflammatory and pro-fibrogenic processes (46). Recent studies have proven the role of Nod-like receptor protein 3 (NLRP3) inflammasome as a key molecule in cell-cell propagation and development of liver fibrosis in MAFLD (47). Again, the NLRP3 inflammasome can be triggered by different ligands, such as PAMPs and DAMPs. Finally, the most significantly studied pathway of regulated necrosis is necroptosis. Necroptosis is featured as passive necrosis, with increased cell volume, swelling of organelles, loss of membrane integrity and cellular collapse, leading to the release of DAMPs (37). This process is initiated by death receptors or PPRs, including TLRs, and needs from RIPK1 and RIPK3 proteins to be executed, among others (48). RIPK3 has emerged as a metabolic regulator and its role as a key manager of metabolism, damage, inflammation, fibrosis and even carcinogenesis has been recently proven (49).

## Immune Cells Interplay in NASH

Body homeostasis is partially dictated through the relationship among the immune cells from all tissues with metabolic activity. Once MAFLD is established, the liver cells will initiate a response in order to control the damage. Among others, the main actions of the liver are detoxification, metabolic activities and key immune roles. Hepatocytes are the most abundant cell type in the liver, reaching up to 70% of the total mass. The rest of hepatic cells are the non-parenchymal cells, comprising hepatic stellate cells, sinusoidal endothelial cells and the immune cell population.

**Figure 2:**
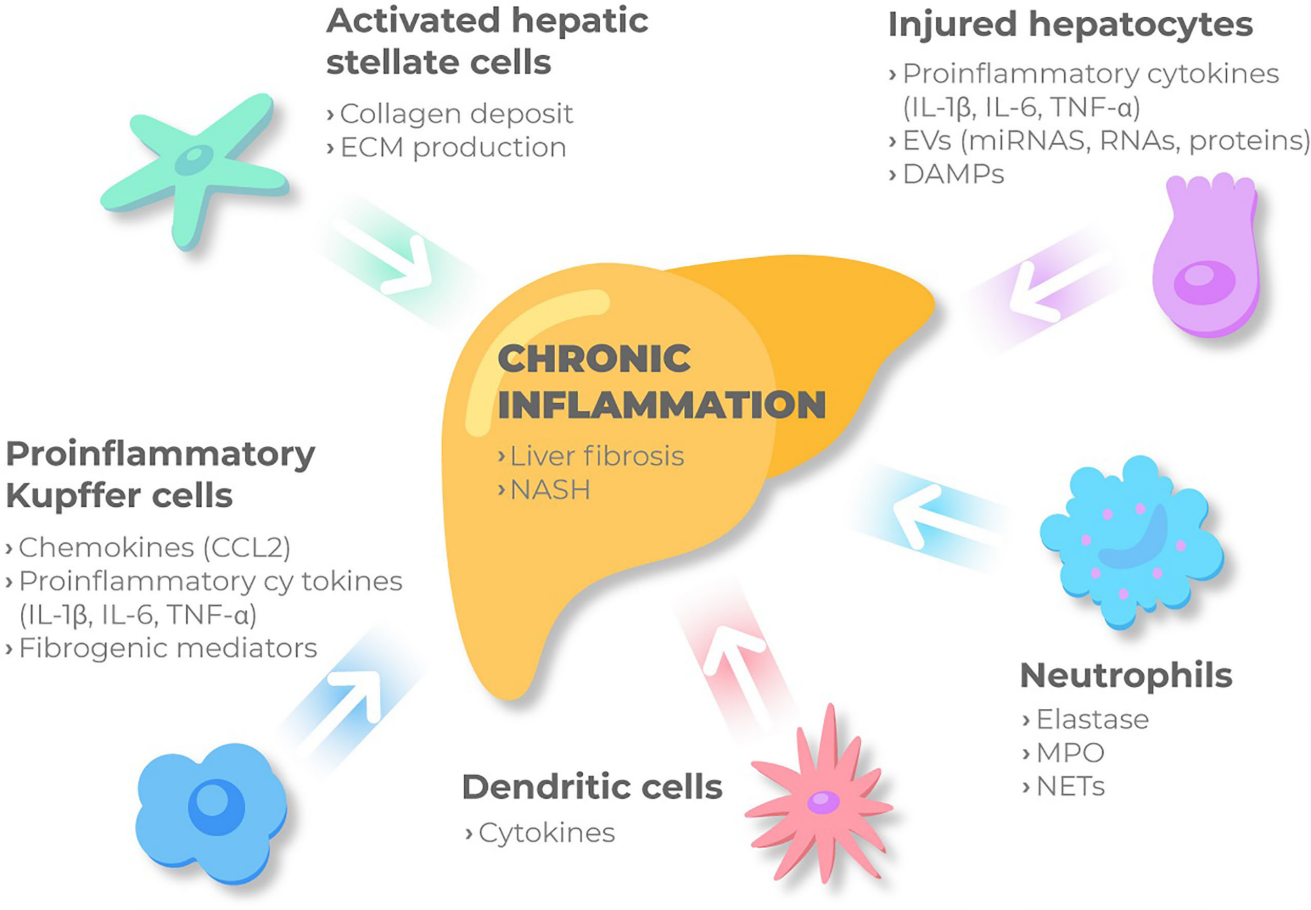
Immune cellular network in MAFLD. Kupffer cells and MoMFs sense the accumulation of inadequate molecules and metabolites in the liver. Then, under these conditions the immune-active phenotype of the liver might be switched on, and KCs are activated releasing chemokines such as CCL2, pro-inflammatory cytokines and fibrogenic mediators. Besides, injured hepatocytes, mainly under metabolic stress, release proinflammatory cytokines, DAMPs and extracellular vesicles to perpetuate chronic inflammation through cell to cell communication. Monocytes and neutrophils are then recruited and activated contributing to inflammation by secreting NETs, elastase and myeloperoxidase. Liver macrophages recruit other immune cells and together with KCs bring out HSC from the quiescent status. Activated HSC will produce ECM and collagen deposit, ultimately promoting liver fibrosis. ECM, extracellular matrix; IL-1β, interleukin 1β; IL-6, interleukin 6; TNF- α, tumor necrosis factor-α; ECM, extracellular matrix; IL-1β, interleukin 1β; IL-6, interleukin 6; TNF- α, tumor necrosis factor-α; EVs, extracellular vesicles; miRNAs, microRNAs; DAMPs, damage-associated molecular patterns; CCL2, C-c motif chemokine ligand 2; NASH, non-alcoholic steatohepatitis; MPO, myeloperoxidase; NETs, Neutrophil extracellular traps.

NASH is characterized by a recruitment of immune cells into the liver, where they release a plethora of inflammatory molecules (**Figure 2**). Some of these cells participate in the whole inflammatory process, such as macrophages, and other just act in specific processes. Particularly, according to differences in activation and function, macrophages can be classified into different subtypes, M1 or classically activated macrophages, with the main function focused on immune surveillance, and M2, the alternative activated macrophages, that downregulate the immune response. Both types have proven to be involved in MAFLD spectrum. Histologically, lobular inflammation is characterized by the enrichment of various immune cells, such as macrophages, monocytes, neutrophils and natural killer cells.

### Resident Macrophages or Kupffer Cells

Macrophages are a heterogeneous population of myeloid-derived mononuclear cells that play a crucial role in the innate immune system. The hepatic macrophage pool consists of liver-resident macrophages or Kupffer cells and monocyte-derived macrophages recruited from the circulation (50). This cell subset is profusely present in healthy livers, facilitating liver homeostasis and being the first-line responders to PAMPs. Liver macrophages are key part of the “second hit”, given their unique location in the liver sinusoid and their ability to recruit inflammatory cells. An increasing amount of studies highlight the role of Kupffer cells or resident macrophages in the transition towards steatohepatitis and liver fibrosis. Indeed, it has been reported that a selective depletion of Kupffer cells ameliorates liver steatosis, insulin resistance and hepatic injury (51). Besides, Kupffer cells act as the primary sensor for both free fatty acids overload and bacterial lipopolysaccharide, integrating these signals and transmitting the information to the hepatocyte, where the accumulation of lipid droplets takes place (52). Latest studies have demonstrated that liver macrophages usually respond to chronic tissue injury by limiting inflammation and achieving endotoxin tolerance, but NASH hinders this protective cell function, leading to a loss of tolerogenicity in liver macrophages and a fail to dampen immune-mediated damage (53). Finally, it has been recently demonstrated that under MAFLD conditions, the number of Kuppfer cells present in the liver is reduced and replaced by macrophages originated in the bone marrow. Recruited macrophages existed in two different subsets, either closely resembling homeostatic KCs or lipid-associated macrophages from obese adipose tissue that expressed Osteopontin, a MAFLD biomarker linked to liver fibrosis (54).

### Monocyte-Derived Macrophages

There are two major types of hepatic macrophages, monocyte-derived and tissue-resident macrophages or Kupffer cells (55). After liver injury, monocytes intrinsically programmed to polarize towards MoMFs migrate from circulation and gather into the liver before getting activated, differentiating into pro-inflammatory macrophages and secreting molecules that contribute to inflammation.

### Neutrophils

Neutrophils are traditionally recognized as part of the first-line defence of the innate immune system, and are continuously released from the bone marrow into peripheral blood under different stimuli, such as proinflammatory cytokines and growth factors, determining their phenotypes. In the recent years, neutrophils have been proposed as key effectors in the transition from simple steatosis to NASH. Indeed, neutrophils infiltration is one of the main features of histological steatohepatitis. Additionally, neutrophils play a key role in regulating the recruitment, activation and programming of other immune cells. The neutrophil-macrophage interaction promotes an inflammatory response by the liver, exacerbating liver inflammation and thereby contributing to NASH progression (56). Among other mechanisms, neutrophils eliminate pathogens through the formation of neutrophil extracellular traps (NETs) (57), which are deconcentrated chromatin structures mixed with inflammatory proteins. NETs modulate immune cells environment and selectively exerts effects on the liver inflammation, and have been recently found elevated in NASH patients and STAM mice (58). Finally, neutrophil elastase was also found to be increased in early stages of MAFLD (59), as well as neutrophil-derived myeloperoxidase (MPO), a molecule that specifically promote NASH-induced liver fibrosis in both animal models and human settings (60).

### Hepatic Dendritic Cells

Liver dendritic cells are immature and tolerogenic in the steady state and acquire a pro-inflammatory phenotype during chronic liver injury. This population, however, appears to be protective, preventing structural injuries (61). This group of cells act as antigen-presenting cells in the liver, although their role is still under debate in NASH development, with contradictory preliminary results. These cells seem to play a role in the lipid storage within the liver, having migratory capacity, and are able to produce particular cytokines acting as a bridge between innate and adaptive processes (62).

### Lymphocytes

Lobular infiltration by lymphocytes is other histological feature of NASH. Besides, in patients with NASH, T-cells and B-cells might form focal aggregates, resembling ectopic lymphoid structures. These structures often develop at sites of inflammation where they can influence the situation (63). Lymphocyte responses contribute to sustained hepatic macrophage activation and natural killer gathering, therefore, interfering with lymphocyte recruitment and activation has proven to ameliorate experimental steatohepatitis in animal models (64). T-helper cells are key regulators of proinflammatory and anti-inflammatory processes, and assist other cells from the immune adaptive system to clear pathogens, such as B-cells, phagocytes and macrophages. Multiple T-cell subsets are involved in MAFLD pathogenesis, exerting differential effects on adiposity, insulin resistance, steatosis, hepatic inflammation, hepatic injury and fibrosis. They can differentiate into several effector types and further characterized by their function and major cytokine secretion (65). These cells can be differentiated mainly into Th1, Th2, Th17 and Th22, and NASH is mainly characterized by an excess of Th1-derived cytokines, such as IL-12 and IFNγ, and a deficiency in Th2-derived cytokines, including IL-4, IL-5 and IL-13 (65). In this regard, as part of the chronic inflammatory process characteristic of this disease, increased IL-17 levels have been previously linked to obesity (66), and hepatic IL-17 signalling pathway has been identified as a contributor in the amelioration of MAFLD following Roux-en-Y gastric bypass surgery (67). Early atherosclerosis has been also associated to an increase in serum IL-17-related chemokine eotaxin, secreted *via* the smooth muscle cells present in the atheromatous vessels in ultrasonography-proven MAFLD patients (68), reinforcing the relationship between cardiovascular diseases and MAFLD. Besides, adipose tissue-derived stem cells seem to prevent liver fibrosis in the context of MAFLD by suppressing IL-17-mediated inflammation in hepatic stellate cells (69). In fact, abolishing the IL-17A axis in preclinical models has been proven to suppress diet-induced obesity by promoting adipose-tissue browning, thermogenesis and energy expenditure (70). Furthermore, this beneficial effect has a direct impact in the liver decreasing NASH and hepatocellular damage (71), suggesting that induction of the IL-17 axis might drive the progression towards more aggressive forms of MAFLD.

### Immune Biomarkers

Multiple immunological mechanisms are involved in MAFLD. Some of them such as the content of extracellular vesicles and some functional single nucleotide polymorphisms contribute to the inflammation, disrupting hepatic homeostasis and leading to worse stages of the disease.

#### Extracellular Vesicles and Non-Coding RNAs

Extracellular vesicles (EVs) are non-nucleated, membrane-bound particles containing lipids, proteins, RNAs and microRNAs. These vesicles are considered a vector of cell-to-cell communication in different situations. They contain different bioactive cargoes depending on their cell of origin, which can exert a variety of actions in the recipient cells. Besides hepatocytes, non-parenchymal cell-derived and extra-hepatic origin EVs are implicated in accelerating the disease progression as there exist inter-cellular and inter-organ crosstalk in MAFLD ^lxi^. Different cell types of the liver secrete as well as receive the cargoes for a plethora of extracellular vesicles, evoking an inflammatory response by the activation of monocytes and macrophages *via* distinct signalling pathways (72). Thus, these particles have been proposed as potential biomarkers or even therapeutic targets for this disease, due to their role in the liver microenvironment, immune response, circulation and the inter-organ crosstalk. It has been recently described that extracellular vesicles in plasma of melanoma patients are partially secreted by residual or relapsing tumor cells, together with the liver and peripheral blood mononuclear cells. This provides new evidence into the innate immune cells defence against tumor and points out the role of EVs as disease biomarkers (73). EVs might contain different molecules, among them, microRNAs can be packaged and released in extracellular vesicles, serving as messengers for cell to cell communication. MicroRNAs are small, non-coding RNAs that are critical regulators for liver physiological processes, regulating gene expression post-transcriptionally by binding mRNA targets and boosting their degradation and/or translational inhibition. There are some microRNAs that are specifically expressed or enriched in certain hepatic cells or in circulation, such as miR-122, miR-194/192, miR-223, miR-21, miR-155 and miR-29 (74). A recent study aiming to determine differences between monocytes and Kupffer cells compared the immunophenotype, gene expression profile, proteome, and microRNAs profiles of both, and found that Kupffer cells expressed a subset of specific miRNAs including mmu-mir-122, mmu-let-7c, mmu-mir-720p, mmu-mir-1224, mmu-mir-2141, and mmu-mir-1944. Further, enrichment analysis of the genes identified as the targets of miRNAs revealed that a number of genes regulated by these elevated miRNAs are involved in signalling pathways associated with the TNFα-induced cascade (75).

#### Single Nucleotide Polymorphisms

Among the different biomarkers, novel single-nucleotide polymorphisms (SNPs) impact on the onset and progression of this disease and are currently under research. The patatin-like phospholipase domain containing 3 (PNPLA3) gene was discovered in a genome-wide association study in 2008, and is recognized as the major gene associated with increased triglyceride content and MAFLD (76). PNPLA3 protein has a triglyceride hydrolase function that is lost in the mutant; therefore patients carrying the risk-allele are more prone to develop NASH and liver fibrosis (77). DIAMOND mice under Western diet with sugar in drinking water that overexpressed the Pnpla3 mutant displayed more steatosis and NASH due to metabolic reprogramming, characterized by increased triglycerides, diglycerides and ceramides and downstream inflammatory pathway activation driving increased stellate cell fibrogenesis (78).

Hydroxysteroid 17-β dehydrogenase 13 (HSD17B13) is a lipid droplet-associated protein that has linked to a variety of liver conditions, such as alcoholic hepatitis (79), Wilson’s disease (80) and MAFLD (81). This has led to clinical trials of anti-HSD17B13 agents in humans, although its deficiency does not reproduce the protective role in animal models as observed in human MAFLD (82).

Liver fibrosis is a core pathologic process shown to be crucial to predict mortality in MAFLD patients. In this regard, interferon lambda 4 (IFNL4) influences innate immunity regulation, and has been established as an aetiology-independent predictor of tissue inflammation and fibrosis (83), and more recently, IFNL4 has been associated with liver fibrosis in MAFLD patients most likely by modulating the activation of innate immunity and necroinflammation (84). In the same line, myeloid-epithelial-reproductive tyrosine kinase (MERTK) gene is expressed by immune and non-immune cells implicated in inflammation. MERTK is mainly expressed in anti-inflammatory M2 macrophages mediating transcriptional changes such as suppression of proinflammatory cytokines and enhancement of inflammatory repressors (85). The variant rs4374383, located in MERTK, has been previously associated with hepatic fibrosis in chronic liver conditions, and its contribution seems to be through the modulation of adipokines, cytokines and circulating mononuclear cells activation in response to fat consumption (86). MERTK role has been explored in a preclinical model, concluding that macrophages enriched in MERTK activate hepatic stellate cells in NASH by inducing TGF-β1 pathway therefore inducing liver fibrosis (87).

Major histocompatibility complex class I-related chain A (MICA) is a ligand for natural killer cells, regulating immune surveillance. MICA SNPs have been previously linked to chronic immune-mediated diseases and liver fibrosis in chronic hepatitis C through TGF-β1-dependent mechanisms (88), and more recently to histological features of NASH and fibrosis (89). In summary, MAFLD has been independently associated with an increased risk of both intrahepatic and extrahepatic events, and related genetic variants amplify its effect on disease outcomes (90).

Finally, IL-6 is a multifunctional cytokine that regulates immune responses, acute phase reactions, haematopoiesis and plays key roles in inflammation, host defence and tissue injury (91). A common variant located in the IL6 gene, rs10499563, was proven to increase susceptibility for NASH, conferring higher risk of hepatic parenchymal damage, including increased ballooning and Mallory bodies, and bridging fibrosis or cirrhosis (92).

## Immune-Mediated Diseases and MAFLD

Most common inflammatory pathways in MAFLD and other immune-mediated diseases depend on the establishment of systemic inflammation. Several epidemiological studies confirmed an increased prevalence of MAFLD in patients with psoriasis, accounting for double the risk of patients without this disease (93), and increasing the extension of psoriasis lesions (94). Psoriasis is a chronic, systemic immune-mediated disease characterized by the development of erythematous, indurated, pruritic plaques in the skin (95). Both entities have a common cytokine-mediated inflammatory background, where the imbalance of pro-inflammatory cytokines and adipokines, including TNF-α, play a key role not just in their pathogenesis, but in MAFLD progression towards NASH. Indeed, in a large, retrospective paediatric cohort study, both, obesity and psoriasis were independent risk factors for the development of metabolic comorbidities and MAFLD, among others, although the relative contribution of obesity was much higher than the one conferred by psoriasis (96). A recent study has reported more than 40% prevalence of MAFLD in a cohort of more than two hundred psoriatic patients, higher than the prevalence described in the general population. This rate was found to be also associated with a higher prevalence of bacterial translocation and a higher pro-inflammatory state, evaluated by increased serum levels of TNF-α and transforming growth factor-beta (TGF-β) (97). Finally, it has been reported that psoriasis plus MAFLD confers a higher risk of developing cardiovascular disease at 10 years compared to psoriasis alone (98). The relationship between other chronic inflammatory skin diseases and MAFLD has also been assessed. A recent study has reported a high prevalence of MAFLD evaluated either by ultrasound or transient elastography in a cohort of hidradenitis suppurativa patients (99).

Regarding to gastrointestinal diseases, inflammatory bowel diseases (IBDs) are featured by a chronic remitting inflammation of the gastrointestinal tract, accounting for Crohn´s disease (CD) and ulcerative colitis (UC). Both entities coexist with MAFLD, especially into the lean-MAFLD population. However, the prevalence reported among IBD patients is still unsettled and variable. Recent meta-analysis concluded that the prevalence of MAFLD among IBD patients was between 27.5% (100) and 32% (101), being in both cases higher than in the general population. MAFLD-related liver malfunction and visceral fat might worsen intestinal inflammation, but the adoption of Mediterranean diet in a CD and UC cohorts was proven to be associated with an improvement of disease activity and inflammatory markers, together with a reduction in hepatic steatosis (102). In a large CD cohort, the relationship with MAFLD and variants in the autophagy-governing immunity-related GTPase M (IRGM) gene was explored, concluding that visceral adipose tissue was directly associated with prevalent MAFLD, being this relationship augmented by functionally annotated IRGM variants (103). Finally, the clinical course of the intestinal disease seems to promote different MAFLD phenotypes, and the more severe IBD is related to more severe steatosis degrees (104).

## Conclusions and Future Perspectives

MAFLD is a leading cause of hepatic transplant worldwide, and its complexity occurs at both the molecular and cellular levels. Nevertheless, therapeutic options are still lacking, mainly due to the multiple unsolved questions regarding its mechanisms. Despite the fact this disease is highly prevalent in the general population, no specific therapies have been approved to date by regulatory agencies. The relationship between this disease and the immune system activation is clear. Inflammation is believed to play a paramount role in the transition of simple steatosis towards non-alcoholic steatohepatitis and more severe forms of hepatic injury, although the exact mechanisms of progression may differ from one patient to another. Yet the hepatic cell-specific role of novel molecules intended to treat NASH and liver fibrosis needs to be deciphered in the context of inflammation.

The number of inappropriate molecules released from liver cells is intimately related, orchestrating both the initiation and propagation of NASH. Therefore, new strategies are under development to regulate the immune system and its interaction with other organs, such as the gut. Finally, the control of liver cell death not just in hepatocytes but in different immune cells is promising and might appear as a potential option to treat severe forms of this disease.

The non-invasive diagnosis of NASH remains as a challenge with especial importance for the monitoring and follow-up of patients, particularly important in those at risk of progressing towards cirrhosis and hepatocellular carcinoma. miRNAs and single-nucleotide polymorphisms appear to have a great potential as non-invasive biomarkers for this condition, correlating with disease severity. These two markers, in conjunction with other molecules released into the bloodstream, might constitute a promising and non-invasive diagnostic tool, the liquid biopsy.

Finally, patients with immune-mediated diseases, such as psoriasis, hidradenitis suppurativa and IBDs should be screened for MAFLD and managed accordingly. It should be considered that some medications for chronic skin and gastrointestinal diseases might have a harmful effect in patients with liver diseases. Multidisciplinary assessment and intense diagnostic efforts in MAFLD patients need to be made and seem to be crucial to ensure the optimal care of these patients. Lastly, a greater understanding of the immune cells heterogeneity, such as macrophages subpopulations, cells which seem to play a key role in the development and progression of MAFLD, will improve novel therapies that will arise in the near future. Novel clinical trials based on immune therapeutic agents following a prior and adequate patient stratification might be expected in the upcoming years.

## Author Contributions

RG-D, RM-V, JA, and MR-G conceived the manuscript. All authors contributed to manuscript revision, read, and approved the submitted version

## Funding

The research leading to these results has received funding from the Consejería de Salud de la Junta de Andalucía under grant agreement PC-0148-2016-0148, the Ministerio de Economía y Competitividad under grant agreement AGL2017-86927-R, the Instituto de Salud Carlos III under grant agreements PI16/01842, PI19/01404, PI19/00589, IFI18/00041 and CD18/00126, and the Fondo Europeo de Desarrollo Regional (FEDER) in cooperation with the Consejería de Salud de la Junta de Andalucía (PE-0451-2018). RG-D has received the Andrew K Burroughs Fellowship from European Association for the Study of the Liver (EASL), the Aprendizaje de Nuevas Tecnologías fellowship from Asociación Española para el Estudio del Hígado (AEEH), and the CIBERehd Grant to support researcher’s mobility.

## Conflict of Interest

The authors declare that the research was conducted in the absence of any commercial or financial relationships that could be construed as a potential conflict of interest.

## Publisher’s Note

All claims expressed in this article are solely those of the authors and do not necessarily represent those of their affiliated organizations, or those of the publisher, the editors and the reviewers. Any product that may be evaluated in this article, or claim that may be made by its manufacturer, is not guaranteed or endorsed by the publisher.

## Abbreviations

MAFLD, metabolic associated fatty liver disease; PAMPs, pathogen-associated molecular patterns; DAMPs, damage-associated molecular patterns (DAMPs); PPRs, pattern recognition receptors; TLRs, toll-like receptors; LPS, lipopolysaccharide; MR, mineralocorticoid receptor; CD, cluster of differentiation; MCD, methionine-choline deficient diet; TREM2, triggering receptor expressed on myeloid cells-2; TNF-α, tumor necrosis factor-α; IL-6, interleukin-6; IL-17, interleukin 17; CK-18, cytokeratin 18; NLRP3, nod-like receptor protein 3; KC, *Kupffer cells;* MoMFs, *Monocyte-derived macrophage; NETs*, neutrophil extracellular traps; MPO, neutrophil-derived myeloperoxidase; EVs, extracellular vesicles; PNPLA3, patatin-like phospholipase domain containing 3; HSD17B13, hydroxysteroid 17-β dehydrogenase 13; IFNL4, interferon lambda 4; MERTK, myeloid-epithelial-reproductive tyrosine kinase; MICA, major histocompatibility complex class I-related chain A; TGF-β, transforming growth factor-beta; IBDs, inflammatory bowel diseases; CD, Crohn´s disease; UC, ulcerative colitis; IRGM, autophagy-governing immunity-related GTPase M; SNPs, single nucleotide polymorphism; IR, insulin resistance; DM2, diabetes mellitus type 2; ECM, extracellular matrix; IL-1β, interleukin 1β; IL-6, interleukin 6; TNF- α, tumor necrosis factor-α.
